# Association of sublingual microcirculation parameters and endothelial glycocalyx dimensions in resuscitated sepsis

**DOI:** 10.1186/s13054-019-2542-2

**Published:** 2019-07-24

**Authors:** Alexandros Rovas, Laura Mareen Seidel, Hans Vink, Timo Pohlkötter, Hermann Pavenstädt, Christian Ertmer, Michael Hessler, Philipp Kümpers

**Affiliations:** 1 Department of Medicine D, Division of General Internal Medicine, Nephrology, and Rheumatology, University Hospital Muenster, Albert-Schweitzer-Campus 1, 48149 Münster, Germany; 2 Department of Anesthesiology, Intensive Care, and Pain Therapy, University Hospital Muenster, Münster, Germany; 3 Department of Physiology, Cardiovascular Research Institute Maastricht, Maastricht University, Maastricht, The Netherlands

**Keywords:** Endothelial glycocalyx, Microcirculation, Perfused boundary region, PBR, Intravital microscopy, Sepsis, Intensive care unit, Sidestream dark field microscopy, Incident dark field illumination imaging, Glycosaminoglycans

## Abstract

**Background:**

The endothelial glycocalyx (eGC) covers the luminal surface of the vascular endothelium and plays an important protective role in systemic inflammatory states and particularly in sepsis. Its breakdown leads to capillary leak and organ dysfunction. Moreover, sepsis-induced alterations of sublingual microcirculation are associated with a worse clinical outcome. The present study was performed to investigate the associations between eGC dimensions and established parameters of microcirculation dysfunction in sepsis.

**Methods:**

This observational, prospective, cross-sectional study included 40 participants, of which 30 critically ill septic patients were recruited from intensive care units of a university hospital and 10 healthy volunteers served as controls. The established microcirculation parameters were obtained sublingually and analyzed according to the current recommendations. In addition, the perfused boundary region (PBR), an inverse parameter of the eGC dimensions, was measured sublingually, using novel data acquisition and analysis software (GlycoCheck™). Moreover, we exposed living endothelial cells to 5% serum from a subgroup of study participants, and the delta eGC breakdown, measured with atomic force microscopy (AFM), was correlated with the paired PBR values.

**Results:**

In septic patients, sublingual microcirculation was impaired, as indicated by a reduced microvascular flow index (MFI) and a reduced proportion of perfused vessels (PPV) compared to those in healthy controls (MFI, 2.93 vs 2.74, *p* = 0.002; PPV, 98.53 vs 92.58, *p* = 0.0004). PBR values were significantly higher in septic patients compared to those in healthy controls, indicating damage of the eGC (2.04 vs 2.34, *p* < 0.0001). The in vitro AFM data correlated exceptionally well with paired PBR values obtained at the bedside (rs = − 0.94, *p* = 0.02). Both PBR values and microcirculation parameters correlated well with the markers of critical illness. Interestingly, no association was observed between the PBR values and established microcirculation parameters.

**Conclusion:**

Our findings suggest that eGC damage can occur independently of microcirculatory impairment as measured by classical consensus parameters. Further studies in critically ill patients are needed to unravel the relationship of glycocalyx damage and microvascular impairment, as well as their prognostic and therapeutic importance in sepsis.

**Trial registration:**

Retrospectively registered: Clinicaltrials.gov, NCT03960307

**Electronic supplementary material:**

The online version of this article (10.1186/s13054-019-2542-2) contains supplementary material, which is available to authorized users.

## Background

The endothelial glycocalyx (eGC) is a negatively charged, carbohydrate-rich layer, lining the luminal surface of the entire vascular endothelium [1, 2]. It is up to 3 μm thick, largely consists of highly sulfated glycosaminoglycans and proteoglycans, and plays a pivotal role in the maintenance of microcirculatory homeostasis [3, 4]. An intact eGC controls capillary permeability, reduces leukocyte-endothelial interactions, mediates shear-induced nitric oxide release, contributes to the regulation of the endothelial redox state, and harbors important anticoagulant mediators [5–7]. Accordingly, the critical importance of the eGC has been highlighted in different vascular pathologies, and particularly in the systemic inflammatory response syndrome (SIRS) and sepsis, where glycocalyx degradation plays a causative role in vascular barrier dysfunction and the development of organ failure, especially lung and kidney injury [8–10].

Observational studies in critically ill patients have shown that the amount of shed eGC constituents in blood samples correlates with sepsis severity and outcome [11–15]. Recently, novel automated acquisition and analysis software (GlycoCheck™, Microvascular Health Solutions Inc., Salt Lake City, UT, USA), able to analyze the perfused boundary region (PBR), an inverse parameter of eGC dimensions in sublingual microvessels, has become available [16]. Pilot studies conducted in the intensive care unit (ICU) revealed that the PBR is indeed markedly increased in critically ill patients compared to healthy controls [3, 17–20]. We were recently able to confirm excellent inter- and intra-observer reproducibility of the GlycoCheck™ method under routine clinical conditions in the emergency room (ER) and ICU [21].

Analysis of the sublingual microcirculation by sidestream dark field (SDF) imaging and incident dark field (IDF) illumination imaging has already been recognized as an interesting tool to improve risk stratification and prognostication and possibly to guide individual therapy in the future [22–27]. For example, De Backer et al. [28] found that the proportion of perfused small sublingual vessels outperformed global hemodynamic variables in the prediction of ICU mortality in patients with severe sepsis. However, whether eGC changes coincide with established parameters of microcirculatory dysfunction in septic patients has not been yet investigated. We therefore measured in vivo, for the first time, eGC dimensions and *classical* microcirculation parameters simultaneously in septic patients. Additionally, we performed further in vitro measurements to evaluate the accuracy of the calculated in vivo PBR values.

## Methods

### Study population and study design

This prospective, observational, cross-sectional study took place from July 2017 to September 2017 in the medical and surgical ICUs of the University Hospital Münster (> 70 ICU beds). The study was performed in accordance with the Declaration of Helsinki, was approved by the competent ethics committee (2016-073-f-S), and was retrospectively registered in Clinicaltrials.gov (NCT03960307).

Thirty adult ICU septic patients, as defined by the Sepsis-3 criteria published by the ESICM-SCCM Sepsis Redefinitions Task Force [29], were enrolled non-consecutively after the initial resuscitation. Written informed consent was obtained from the patients or their legal representatives. Exclusion criteria were underage, pregnancy, oral mucosal inflammation, or injury, which could locally influence the sublingual microvasculature. Ten apparently healthy volunteers served as controls.

Demographic variables, routine chemistry tests, and physiological parameters, including the Sequential Organ Failure Assessment (SOFA) score [29] and a contemporary version of the Charlson Comorbidity Index (CCI) [30], were obtained for each subject immediately before the sublingual videomicroscopy (Table 1). Serum samples from patients and controls were obtained and immediately centrifuged at 4 °C with 4000*g* for 10 min and stored at − 80 °C for further analysis of the glycocalyx components.

**Table 1:** Baseline characteristics

Variable	Healthy individuals	Septic patients	*p* value
Number of participants (*n* (%))	10	30	–
Female sex (*n* (%))	5 (50)	7 (23)	0.11
Age (years, median (IQR))	30 (27–34)	67 (58–80)	< 0.0001
BMI (kg/m^2^, median (IQR))	23.6 (21.3–26.1)	25.3 (21.7–28.2)	0.34
Duration of sepsis at study inclusion (days, median (IQR))*	–	2 (1–5)	–
SOFA score (median (IQR))	–	9 (5–12)	–
Organ replacement therapy (*n* (%))	–	18 (60)	–
Mechanical ventilation (*n* (%))	–	17 (56.7)	–
Acute dialysis (*n* (%))	–	5 (16.7)	–
Vasopressors (*n* (%))	–	20 (66.7)	–
Norepinephrine dose (μg/kg/min)	–	0.05 (0–0.16)	–
Septic shock (*n* (%))**	–	3 (10)	–
Hospital mortality (*n* (%))	–	10 (33.3)	–
CCI score (median (IQR))	–	1.5 (0.8–2.3)	–
Comorbidities (*n* (%))			
Chronic respiratory disease	–	8 (26.7)	–
Congestive heart failure	–	16 (53.3)	–
Chronic hepatic disease	–	3 (10)	–
Dialysis-dependent CKD	–	1 (3.3)	–
Malignancy	–	5 (16.7)	–
Diabetes mellitus	–	5 (16.7)	–
Focus of infection (*n* (%))			
Respiratory tract	–	17 (56.7)	–
Prosthesis	–	4 (13.3)	–
Gastrointestinal tract	–	3 (10)	–
Unknown	–	2 (6.7)	–
Skin	–	2 (6.7)	–
Urinary tract	–	1 (3.3)	–
Heart	–	1 (3.3)	–
Endothelial glycocalyx (median (IQR))			
PBR 5–25 (μm)	2.04 (1.97–2.10)	2.34 (2.21–2.46)	< 0.0001
Syndecan-1 (ng/ml)	21.3 (13.2–56.7)	204.5 (114.2–358.9)	< 0.0001
Microcirculation data (median (IQR))			
TVD (mm/mm^2^)	18.88 (17.56–21.68)	19.17 (17.06–20.24)	0.87
PVD (mm/mm^2^)	18.54 (17.19–21.26)	16.97 (14.96–19.87)	0.08
PPV (%)	98.73 (96.55–99.80)	92.58 (85.63–97.14)	0.0004
MFI (points)	2.93 (2.89–2.96)	2.74 (2.58–2.91)	0.002
HI (%)	0.04 (0.01–0.06)	0.08 (0.03–0.16)	0.04
Macrocirculation data (median (IQR))			
MAP (mmHg)	94.2 (85.4–102.4)	73.0 (67.5–84.6)	0.0001
Heart rate (pulse/min)	73 (65–83)	91 (81–101)	0.0004
Respiratory rate (breaths/min)	14 (13–15)	20 (17–26)	< 0.0001
Temperature (°C)	36.6 (36.5–36.8)	37 (36.4–37.7)	0.12
Laboratory data (median (IQR))			
CRP (mg/dl)	0.5	23.2 (16.9–33.3)	< 0.0001
IL-6 (ng/ml)	2.0 (2.0–2.5)	367.0 (96.0–1121.0)	< 0.0001
PCT (ng/ml)	0.04 (0.03–0.05)	9.08 (1.24–48.23)	< 0.0001
pH	–	7.42 (7.36–7.48)	–
Lactate (mmol/l)	0.90 (0.65–1.20)	1.70 (0.98–2.00)	0.001
Albumin (g/dl)	4.8 (4.5–5.0)	2.5 (2.0–3.0)	< 0.0001
Total serum protein (g/dl)	7.1 (6.8–7.5)	5.5 (5.1–6.0)	< 0.0001

*p* value was calculated between healthy individuals and ICU patients

*BMI* body mass index, *CCI score* Charlson Comorbidity Index score, *CKD* chronic kidney disease, *CNS* central nervous system, *CRP* C-reactive protein, *HI* heterogeneity index, *IL-6* interleukin-6, *IQR* interquartile range, *MAP* mean arterial pressure, *MFI* microvascular flow index, *PBR* perfused boundary region, *PCT* procalcitonin, *PPV* proportion of perfused vessels, *PVD* perfused vessel density, *RBC* red blood cell, *SOFA score* Sequential Organ Failure Assessment score, *TVD* total vessel density, *WBC* white blood cell

*n = 9 of 30 patients (30%) were included within 24 h after fulfilling Sepsis-3 criteria

**Septic shock: vasopressors required to maintain MAP ≥ 65 mmHg and serum lactate > 2 mmol/l.

The subsequent assessment of the microcirculation and the eGC was independently performed in random order by two physicians (AR, LMS), as described in detail below. Both physicians were experienced in these techniques and trained to recognize and avoid pressure and movement artifacts. The real-time assessment of microvascular flow index (MFI by “eyeballing”) was used as a post hoc verification to ensure representative recordings of the microvasculature [31]. The different parameters assessed by the two methods are summarized in Additional file 1: Table S1.

### Assessment of the endothelial glycocalyx in vivo

The endothelial glycocalyx was assessed sublingually using the GlycoCheck™ software, coupled with a stroboscopic, SDF camera (CapiScope HVCS, KK Technology, Honiton, UK) by a physician experienced in the method (AR).

The software automatically detects, records, and analyzes the microvessels with diameters between 5 and 25 μm. Specifically, it calculates the dynamic lateral movement of the red blood cells (RBCs) into the permeable part of the eGC layer, expressed as the PBR (in μm). An impaired eGC permits a greater number of RBCs to penetrate deep into the endothelium, which is translated as an increase in the PBR value [16].

Briefly, the GlycoCheck™ software allows video acquisition after predefined image quality criteria (motion, intensity, and focus) are fulfilled. Each complete measurement consists of at least 10 5-s videos (40 frames/video), containing a total of about 3000 vascular segments of 10 μm each. All videos are deliberately obtained from different positions in the sublingual microvasculature. The software automatically subjects the vascular segments obtained to a strict quality check. After marking and discarding invalid segments, the software obtains up to 840 radial intensity profiles for each valid vascular segment and, based on the presence of RBCs, calculates the RBC filling percentage, signal quality, and RBC column width (RBCW). The distribution of RBCs in each valid segment defines the median RBCW, as well as the outer edge of the RBC-perfused lumen (Dperf). The PBR is defined as the distance between the RBCW and the outer edge of the Dperf and is calculated using the following formula: (Dperf − RBCW)/2. The software classifies the PBR values to their corresponding RBCW (5–25 μm), presents a median PBR for each vessel diameter category (5–9 μm, 10–19 μm, and 20–25 μm), and provides a single, average, weighted PBR value (5–25 μm) for each measurement. Data from two complete measurements (hereafter referred to as “measurement set”) were manually averaged to avoid sampling error and to counterbalance spatial heterogeneity of the sublingual microcirculation. The excellent inter- and intra-observer reproducibility under real-life conditions has been previously reported by our group [21].

### Assessment of sublingual microcirculation

The microcirculation was visualized sublingually with the use of an IDF illumination hand-held vital microscope (CytoCam™, Braedius Medical BV, Huizen, The Netherlands) by a physician experienced in the method (LMS) [32]. At least five videos of the sublingual microcirculation were recorded from different positions in the sublingual region. The videos obtained went through a quality check, based on the recommendations of Massey et al. [33], and were manually discarded if necessary. The remaining 116 high-quality videos were semi-manually analyzed offline by an experienced operator (MH) blinded for the patients’ clinical data, as thoroughly described previously [34], using dedicated software (Capillary Mapper 1.4, University of Muenster Medical Centre, Münster, Germany [35]). Total vessel density (TVD), perfused vessel density (PVD), proportion of perfused vessels (PPV), microvascular flow index (MFI), and heterogeneity index (HI) were calculated in the microvessels under 20 μm, based on current recommendations, in order to assess microcirculation abnormalities in critically ill patients [25], as described in detail previously [36].

### Quantification of circulating syndecan-1 levels

The proteoglycan syndecan-1 (syn-1), an important core protein of the eGC, was measured in serum using a commercially available enzyme-linked immunosorbent assay (ELISA) (Gen-Probe Diaclone Research, Besançon, France). The measurements were performed in duplicate according to the manufacturer’s instructions and, at the same time, by the same investigator (AR), blinded to individuals’ characteristics.

### Assessment of endothelial glycocalyx in vitro

#### Cell culture

The human umbilical vein endothelial cell (EC) line EA.hy926 was grown on glass coverslips (Roche Diagnostics GmbH, Mannheim, Germany) for at least 3 days until confluence in Dulbecco’s modified Eagle’s medium (DMEM, Invitrogen, Karlsruhe, Germany) supplemented with 10% fetal bovine serum (FBS, FBS Superior Biochrom, Berlin, Germany) and 1% penicillin/streptomycin (Biochrom, Berlin, Germany) in a 5% CO_2_-enriched environment at 37 °C.

#### Atomic force microscopy

The thickness of the eGC in vitro was determined using the atomic force microscope (AFM) nanoindentation technique, as described previously in detail [37–39]. Briefly, cells were analyzed in 4-(2-hydroxyethyl)-1-piperazineethanesulfonic acid (HEPES) buffer (140 mM NaCl, 5 mM KCl, 1 mM CaCl2, 1 mM MgCl2, 5 mM glucose, 10 mM HEPES) supplemented with 1% FBS at 37 °C in a fluid chamber with a Nanoscope III Multimode AFM (Veeco, Mannheim, Germany). A triangular cantilever (Novascan Technologies, Boone, IA, USA) with a mounted spherical tip (diameter 10 μm) and a spring constant of 10 pN/nm periodically indents the cells. A laser beam is used to quantify the cantilever deflection. Once the force acting on the cantilever, the piezo displacement, and the deflection sensitivity are known, the thickness of the eGC can be calculated. A more detailed description of the AFM method is provided in Additional file 1: Figure S1.

### Statistical analysis

Data are presented as absolute numbers, percentages, means with standard deviations, or medians with corresponding 25th and 75th percentiles (interquartile range (IQR)), as appropriate. The non-parametric Mann-Whitney *U* test and the chi-square test were used to compare the parameters between the groups. Agreement between MFIs obtained by eyeballing was visualized by the Bland-Altman method. Spearman rank correlation coefficient was used to assess the correlations between variables. Associations between PBR and microcirculatory parameters were evaluated using multiple linear regression. All the tests used were two-sided, and statistical significance was set at *p* < 0.05. Our study was powered to detect a moderate correlation (Spearman correlation coefficient = 0.5) between PBR and the microcirculation parameters in the septic cohort with 80% power given a two-sided alpha of 0.05 [40]. SPSS version 24 (IBM Corporation, Armonk, NY, USA) and GraphPad Prism version 7 (GraphPad Prism Software Inc., San Diego, CA, USA) were used for statistical analyses and preparation of figures.

## Results

The clinical and demographic characteristics of the 40 study participants are shown in Table 1. Approximately 55% of the patients had a respiratory focus of infection, while the remainder demonstrated other etiologies. From a total of 30 septic patients, 18 (60%) required organ replacement therapy and 20 (66.67%) were vasopressor-dependent at inclusion in the study. Our sepsis cohort had a median (IQR) SOFA score of 9 (5–12), indicating moderate disease severity. Nine (30%) patients were included during the first 24 h of sepsis. However, this subgroup did not differ from patients recruited thereafter (Additional file 1: Table S2).

**Fig. 1:**
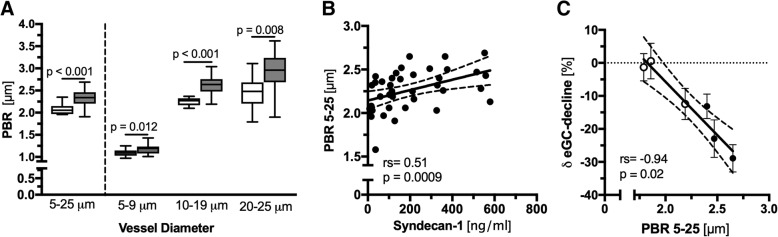
Endothelial glycocalyx dimensions measured in vivo and in vitro. **a** Boxplots of PBR values of healthy controls (white) and septic patients (grey) based on the different microvascular diameter ranges. **b** Correlation of sublingually measured PBR and paired syndecan-1 values. **c** A subpopulation from **a** was randomly selected, and ECs were incubated with 5% sterile-filtered human serum from three septic patients (black circles) and three apparently healthy individuals (white circles), respectively. Scatter plot showing the association between AFM-derived eGC decline (in vitro) and corresponding PBR values (in vivo). Each circle represents the mean of three independent experiments (consisting of ≥ 5 indentation curves in each of ≥ 10 cells) for each individual serum. Incubation without human serum served as control. Data are presented as mean ± SEM. AFM, atomic force microscopy; eGC, endothelial glycocalyx; ICU, intensive care unit; PBR, perfused boundary region; SEM, standard error of mean

The sublingual glycocalyx assessment revealed significantly higher PBR values in septic patients compared to those in healthy controls (2.04 μm [95% CI 1.97 to 2.1] vs 2.34 μm [95% CI 2.21 to 2.46], *p* < 0.0001). This difference was present across all three predefined microvascular diameter categories (Fig. 1a). Circulating levels of the glycocalyx core protein syn-1 were about tenfold higher in septic patients compared to the levels in controls (204.5 ng/ml [95% CI 114.2 to 358.9] vs 21.3 ng/ml [95% CI 13.2 to 56.7], *p* < 0.0001) and correlated moderately with PBR values (rs = 0.51 [95% CI 0.22 to 0.72], *p* = 0.0009; Fig. 1b).

Moreover, we exposed ECs to sterile-filtered, randomly selected sera (5%; diluted in buffer) from three septic patients and three apparently healthy donors for 24 h. The delta changes of eGC thickness measured in vitro correlated strongly with paired PBR values obtained at the bedside (rs = − 0.94, *p* = 0.02, Fig. 1c and Additional file 1: Figure S2).

**Fig. 2:**
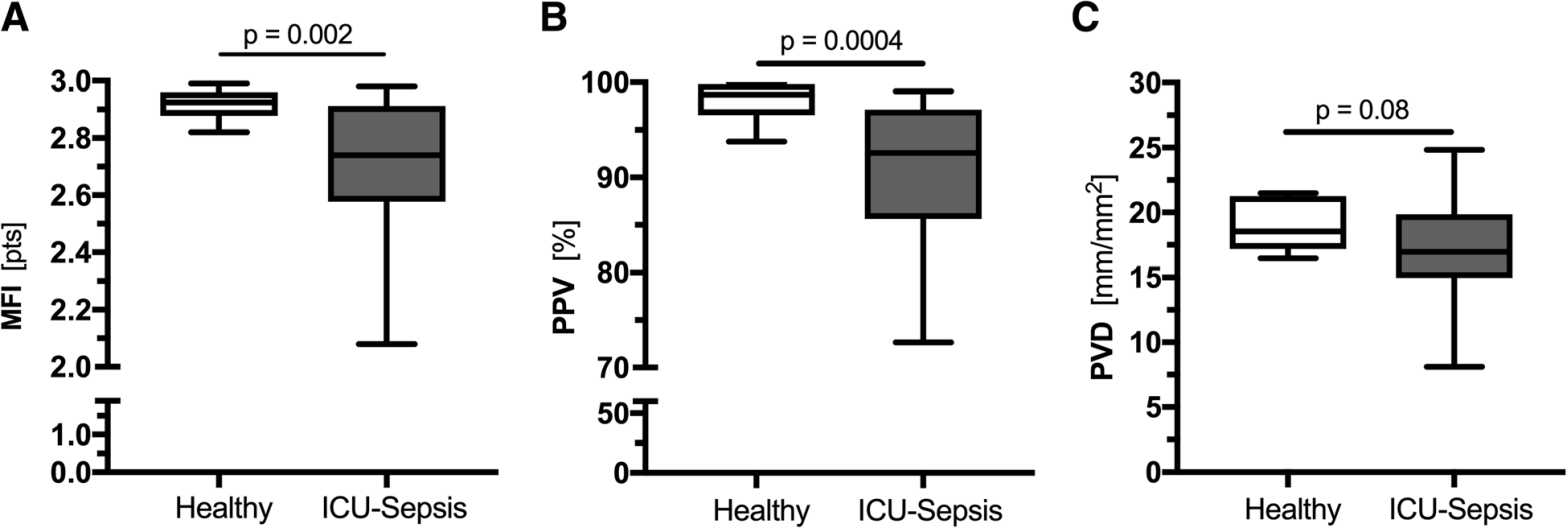
Boxplots of microcirculation parameters in healthy controls and septic patients. **a** MFI and **b** PPV values revealed a damaged microcirculation in the ICU septic population. **c** The measured PVD tended to be lower in septic patients compared to the healthy controls. ICU, intensive care unit; MFI, microvascular flow index; PPV, proportion of perfused vessels; PVD, perfused vessel density

Analysis of IDF videos showed that among the different parameters of microcirculatory perfusion, the MFI (2.74 points [95% CI 2.58 to 2.91] vs 2.93 points [95% CI 2.89 to 2.96], *p* = 0.002) and the PPV (92.58% [95% CI 85.63 to 97.14] vs 98.73% [95% CI 96.55 to 99.80], *p* = 0.0004) were significantly lower in septic patients compared to the controls (Fig. 2a, b). The PVD tended to be lower in septic patients (16.97 mm/mm^2^ [95% CI 14.96 to 19.87] vs 18.54 mm/mm^2^ [95% CI 17.19 to 21.26], *p* = 0.08, Fig. 2c), whereas the TVD was no different between the two groups (19.17 mm/mm^2^ [95% CI 17.06 to 20.24] vs 18.88 mm/mm^2^ [95% CI 17.56 to 21.68], *p* = 0.87). Neither PBR nor MFI and PPV were related to age, sex, or comorbidities (Additional file 1: Table S3).

In a pooled analysis of the measurements from all 40 study participants, PBR and microcirculation parameters (MFI and PPV) were correlated with several markers of critical/acute illness (Table 2), including mean arterial pressure (MAP), C-reactive protein (CRP), interleukin-6 (IL-6), and procalcitonin (PCT) levels, as well as SIRS and SOFA scores. Nevertheless, no association between PBR and microcirculatory parameters could be revealed, even after adjusting for important demographic and clinical variables (Additional file 1: Table S4).

**Table 2:** Significant correlations of PBR, MFI, and PPV with laboratory data and acute/critical illness scores

Variable	PBR	MFI	PPV
CCI (points)	0.39 (0.08 to 0.63)*	–	–
CRP (mg/dl)	0.54 (0.26 to 0.74)***	− 0.59 (− 0.76 to − 0.32)***	− 0.61 (− 0.78 to − 0.35)***
Heart rate (pulse/min)	–	− 0.41 (− 0.64 to − 0.10)**	− 0.46 (− 0.68 to − 0.16)**
IL-6 (ng/ml)	0.45 (0.14 to 0.68)**	–	–
MAP (mmHg)	− 0.37 (− 0.62 to − 0.06)*	–	0.33 (0.01 to 0.58)*
PCT (ng/ml)	0.37 (0.37 to 0.62)*	–	–
SIRS score (points)	0.44 (0.14 to 0.67)**	–	− 0.41 (− 0.65 to − 0.11)**
SOFA score (points)	0.44 (0.14 to 0.67)**	–	–
Total serum protein (g/dl)	− 0.39 (− 0.64 to − 0.06)*	0.53 (0.23 to 0.73)***	0.50 (0.20 to 0.72)**

Spearman correlation was used

*CRP* C-reactive protein, *IL-6* interleukin-6, *MAP* mean arterial pressure, *MFI* microvascular flow index, *PBR* perfused boundary region 5–25 μm, *PCT* procalcitonin, *PPV* proportion of perfused vessels, *SOFA score* Sequential Organ Failure Assessment score

*p < 0.05, **p < 0.01, ***p < 0.001

**Fig. 3:**
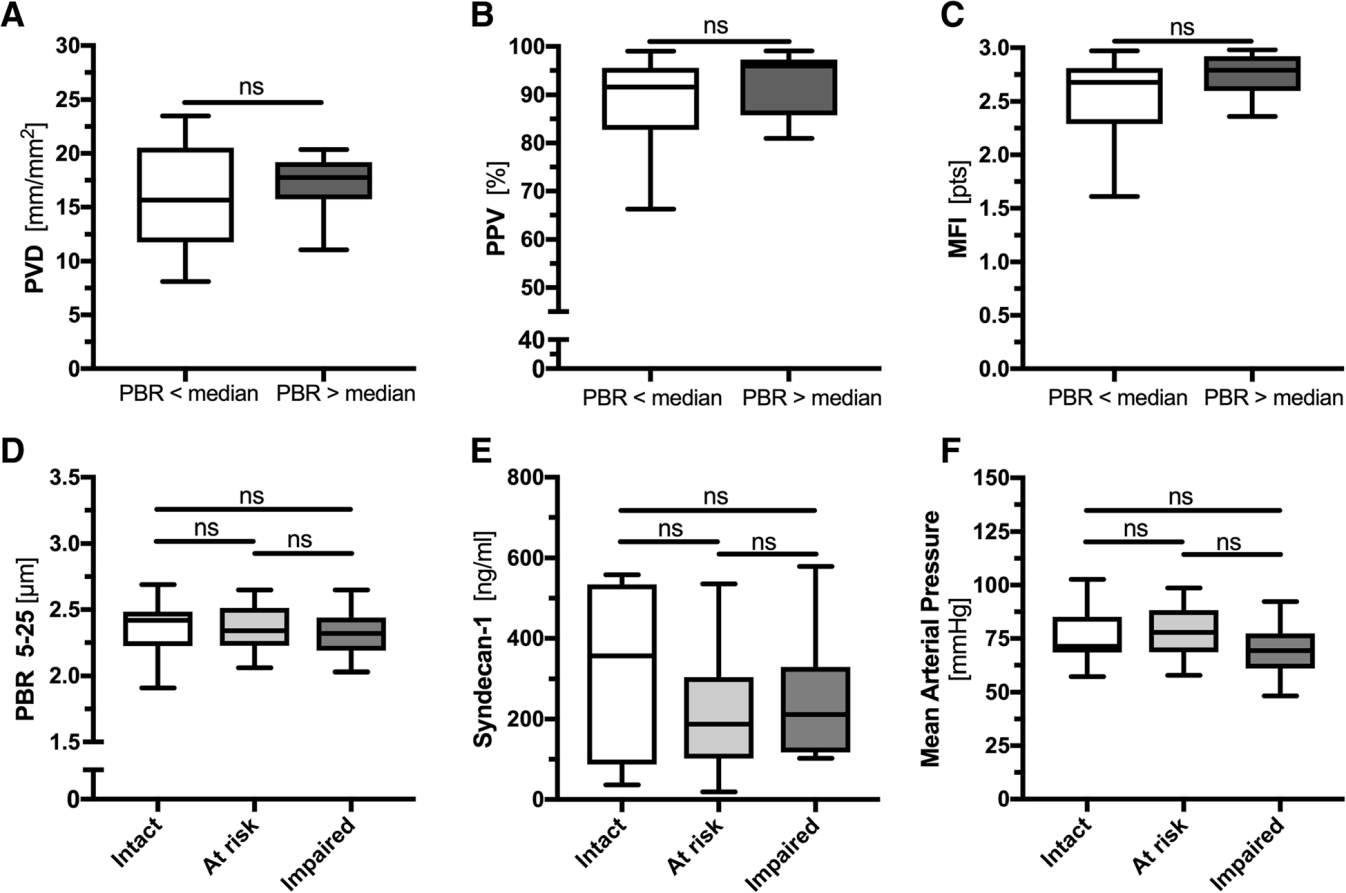
Association of microcirculation and endothelial glycocalyx parameters. **a**–**c** Boxplots of PVD, PPV, and MFI values after dichotomizing the sepsis group by the median PBR values. **d**–**f** Boxplots of septic patients classified on the basis of their microcirculation parameters into the following groups: “intact” (PPV > 90% and MFI > 2.9), “at risk” (PPV ≤ 90% or MFI ≤ 2.9), and “impaired” (PPV ≤ 90% and MFI ≤ 2.9). No difference was observed between the groups regarding PBR, syn-1 levels, or MAP. ICU, intensive care unit; MAP, mean arterial pressure; MFI, microvascular flow index; PBR, perfused boundary region; PPV, proportion of perfused vessels; PVD, perfused vessel density; syn-1, syndecan-1

Neither PBR values nor syn-1 levels correlated with any of the microcirculatory parameters in the sepsis cohort (Additional file 1: Table S5 and Figure S3). This lack of association persisted even when we dichotomized the sepsis group by the median PBR value (Fig. 3a–c). In a different approach, we classified the patients based on their microcirculation parameters into the following three groups: “intact” (PPV > 90 and MFI > 2.9), “at risk” (PPV ≤ 90 or MFI ≤ 2.9), and “impaired” (PPV ≤ 90 and MFI ≤ 2.9). Again, no difference was observed between the groups regarding PBR, syn-1 levels, or MAP (Fig. 3d–f).

## Discussion

To the best of our knowledge, this is the first study specifically investigating the associations between the eGC dimensions and established parameters of the sublingual microcirculation in patients with sepsis. The main results of the current study revealed that, although both microcirculation and glycocalyx parameters correlated plausibly with markers of acute/critical illness, no association could be established between the eGC thickness and the microcirculation data obtained (TVD, PVD, PPV, MFI, HI). Furthermore, we investigated, for the first time, the accuracy of the calculated in vivo PBR. This correlated well with circulating syn-1, a marker for glycocalyx shedding, and was in good agreement with glycocalyx thickness measured by AFM, a method that can detect even the modest changes in the nanomechanics of the eGC [37].

The observed dissociation between PBR dimensions and microvascular perfusion in sepsis has already been indicated by two earlier interventional studies examining the effects of leuko-depleted RBC transfusions or activated protein C, respectively [19, 20]. In both studies, median PBR values remained unchanged while an improvement in microvascular parameters was seen in the treatment groups. In contrast, two longitudinal studies conducted in patients undergoing cardiac surgery suggested the existence of an association between the alterations in microcirculation parameters and eGC dimensions [18, 41]: Koning et al. assessed the sublingual microcirculation and eGC dimensions perioperatively in 36 patients undergoing cardiac surgery with or without cardiopulmonary bypass (CPB) [18]. The CPB procedure increased the PBR and decreased the PVD, whereas off-pump surgery decreased the PBR without affecting the PVD. Dekker et al. analyzed the sublingual microvasculature in 17 patients undergoing non-pulsatile CPB [41]. They found a small increase in PBR and a sustained decrease in PVD and PPV during the 72-h follow-up. Although PBR values during CPB correlated moderately with shed levels of the glycosaminoglycan heparan sulfate, another major component of the glycocalyx, no correlation between PBR and classical parameters of the microcirculation was found. It appears that the reported association between the eGC dimensions or shedding products and microcirculation parameters reflects CPB-induced concordant longitudinal changes, rather than true correlations between the parameters at single time points.

Clinical studies monitoring the sublingual microcirculation during resuscitation procedures revealed the existence of a dissociation between micro- and macrocirculation in septic patients [28, 42]. This phenomenon was recently termed “loss of hemodynamic coherence” [42–45]. In line with these studies, we did not observe any correlation of either SOFA score or MAP with MFI. It is intriguing, however, that this dissociation seems to be absent for the eGC, since eGC dimensions did correlate plausibly with parameters of disease severity and macrocirculation. Our results strengthen the notion that microvascular perfusion and eGC dimensions are two differentially regulated entities, which do not inevitably show concordant changes. In other words, not all patients with an impaired microcirculation exhibit a damaged eGC, and vice versa. The challenging question is why is that? It is conceivable that several factors play a role.

First, we have learned from preclinical murine studies that pulmonary glycocalyx loss develops within 8 h after the induction of polymicrobial abdominal sepsis [46], whereas injection of *Escherichia coli* lipopolysaccharides (LPS) results in overt glycocalyx damage within 30 min [9]. In human endotoxemia, sublingual eGC thickness was lower at 4 h after LPS administration [47]. In summary, a likely explanation would be that the threshold for, as well as the onset of, eGC damage in human sepsis is diverse, leading to spatiotemporal uncoupling of the two entities.

Second, previous studies have shown that, despite the loss of hemodynamic coherence in individual patients, the classical parameters of sepsis-induced microcirculatory dysfunction respond to resuscitation and optimization of arterial pressure in general [24, 28, 48]. Consistent with this notion, De Backer et al. reported that microcirculatory alterations are less severe in the later phase of sepsis than in the earlier phase [28]. Microcirculatory and PBR derangements in our resuscitated sepsis cohort (only 10% in shock) were comparable to values in ICU patients with moderate disease severity reported by other groups [19, 21, 27, 49]. Data regarding the regeneration of the eGC in both human and murine sepsis are missing. Cell culture experiments indicate that after enzymatic degradation, full recovery of the eGC requires at least 3 days under normal (i.e., non-septic) in vitro conditions [50, 51]. Although extrapolation of this finding to the ICU is not valid, it is conceivable that regeneration of the eGC in our patients may considerably lag behind the successful stabilization of the macro- and microcirculation. What makes it even more complicated is the possibility that targeted interventions intended at improving the macrocirculation—for example, the correction of hypovolemia [26]—may augment or even induce glycocalyx degradation, if performed excessively [8, 52].

Third, another explanation could be that the two entities, microcirculation and eGC, are controlled by different regulatory and compensatory mechanisms, including hormonal, neural, biochemical, and vascular control systems [53–56]. We have recently shown, for example, that the activational state of the endothelial-specific Tie2 receptor controls glycocalyx damage in a non-redundant fashion [39]. However, the Tie2 receptor is not expressed by vascular smooth muscle cells and has no direct effect on vasomotor tone.

We acknowledge some limitations in our study. First, it is a single-center study with a limited sample size. Nevertheless, we could not detect *any* trend pointing to an association between classical microcirculation parameters and eGC dimensions. The reason for the small number of serum samples used for the in vitro experiments is that the AFM technique is time-consuming and sophisticated, which precludes the analysis of a considerably larger random sample. Further, it is possible that our AFM approach, in the absence of fluid shear stress and/or reduced abundance of plasma proteins, can only detect thickness in denser glycocalyx layers close to the plasma membrane, thereby potentially underestimating the “fluffier” outer (bloodstream-oriented) regions of the eGC [57, 58]. Second, we had to use two separate camera systems to visualize the sublingual microvasculature and thus cannot exclude a sampling error. However, the fact that the real-time assessment of MFI by eyeballing showed good agreement between the two subsequent videomicroscopic examinations (Additional file 1: Figure S4) argues against this hypothesis. Third, we cannot exclude that the use of two different techniques (SDF, IDF) might contribute to the observed dissociation. IDF has a better definition and magnification and can detect a larger number of vessels than SDF. However, as the GlycoCheck™ system can reliably detect and analyze RBC flux (and thus calculate the PBR) even in the capillaries down to a size of 4 μm, this difference is probably negligible. Furthermore, the absence of any correlation between microcirculatory and eGC parameters (both PBR and syndecan-1) supports our results. Fourth, our study had a cross-sectional design and was set up neither to detect causality of eGC or microcirculation alterations nor to test the performance of PBR and microcirculation parameters for the outcome prediction. To clarify these issues, we initiated two prospective, observational, longitudinal studies to evaluate the eGC dimensions together with microcirculation analysis in the ER (Early Detection of x Damage in Emergency Room Patients—the EDGE Study, Clinicaltrials.gov Identifier: NCT03126032) and in the ICU (Analysis of Sublingual Glycocalyx Damage at ICU Admission to Predict Risk of Death—the ASGARD Study, Clinicaltrials.gov Identifier: NCT03847493).

## Conclusions and outlook

In summary, our data indicate an uncoupling between eGC and microcirculatory parameters in resuscitated sepsis. The mechanism behind this observed dissociation remains unclear. Therefore, future experimental and clinical studies are needed to unravel the relationship of glycocalyx damage and microvascular impairment, as well as their prognostic and therapeutic importance in sepsis.

## Additional file

Additional file 1: **Table S1.** Summary of microcirculation and endothelial glycocalyx parameters [16, 25]. **Figure S1.** Analysis of the endothelial glycocalyx (eGC) in cell culture via atomic force microscopy (AFM). Surface approach: the cantilever (AFM tip) approaches the sample surface vertically. The reflection of a laser beam from the back of the cantilever is continuously detected by a photodiode. First slope: reaching the surface, the cantilever, serving as a soft spring, is deflected while indenting into the sample. The changing laser beam reflection is plotted as a function of sample position along the *z*-axis. By including the cantilever’s spring constant and the optical lever sensitivity, a force-versus-indentation curve can be generated to provide information about how much force (in pN) is needed to indent a certain distance (in nm) into the sample. The first slope of the curve reflects the indentation of the eGC. Second slope: in the second slope, more force is needed to indent into the surface, which reflects the cell cortex with the plasma membrane and actin web. Due to the linearity of the first slope, a regression line can be generated manually through the starting points of both slopes using PUNIAS (protein unfolding and nano-indentation analysis software, version 1.0, release 2.1, http://punias.free.fr). Projected to the *x*-axis, the distance between both starting points represents the thickness of the eGC [38, 39]. **Table S2.** Baseline characteristics of septic patients stratified for sepsis duration. **Figure S2.** Endothelial glycocalyx dimensions measured in vivo and in vitro. Scatter plot showing the association between AFM-derived eGC thickness (in vitro) and corresponding PBR values (in vivo) in three apparently healthy individuals (white circles) and in three septic patients (black circles). Each circle represents the mean of three independent experiments (consisting of ≥ 5 indentation curves in each of ≥ 10 cells) for each individual serum. Incubation without human serum served as control. Data are presented as mean ± SEM. **Table S3.** Correlations of PBR, PPV, MFI, age, sex, and comorbidities in the septic cohort. Spearman correlation was used. The *p* values are indicated in brackets. **Table S4.** Simple and multiple linear regression (PBR as dependent variable). Dependent variable: PBR. **Table S5.** Correlations of microcirculatory and eGC parameters in the septic cohort. Spearman correlation was used. The *p* values are indicated in brackets. **Figure S3.** Correlations of microcirculation and endothelial glycocalyx parameters. (A–C): Correlation of PBR with PVD, PPV, and MFI. (D–F): Correlation of syndecan-1 with PVD, PPV, and MFI. ** Figure S4.** MFI (eyeballing) obtained in real-time with the two different systems. (A): Correlation between the MFI (eyeballing) values obtained at the bedside. (B): Bland-Altman plot showing the limits of agreement (bias ± 1.96 SD) between paired MFI values for the Cytocam and GlycoCheck™ system (eyeballing). One point can represent more than one individual. (DOCX 606 kb)

## Data Availability

The datasets used and/or analyzed during the current study are available from the corresponding author on reasonable request.

## Abbreviations

AFM: Atomic force microscopy/microscope
BMI: Body mass index
CCI: Charlson Comorbidity Index
CKD: Chronic kidney disease
CNS: Central nervous system
CPB: Cardiopulmonary bypass
CRP: C-reactive protein
EC: Endothelial cell
eGC: Endothelial glycocalyx
ELISA: Enzyme-linked immunosorbent assay
ER: Emergency room
FBS: Fetal bovine serum
HI: Heterogeneity index
ICU: Intensive care unit
IDF: Incident dark field
IL-6: Interleukin-6
IQR: Interquartile range
LPS: Lipopolysaccharides
MAP: Mean arterial pressure
MFI: Microvascular flow index
PBR: Perfused boundary region
PCT: Procalcitonin
PPV: Proportion of perfused vessels
PVD: Perfused vessel density
RBC: Red blood cell
RBCW: Red blood cell column width
SD: Standard deviation
SDF: Sidestream dark field
SEM: Standard error of mean
SIRS: Systemic inflammatory response syndrome
SOFA: Sequential Organ Failure Assessment
syn-1: Syndecan-1
TVD: Total vessel density
WBC: White blood cell
