# Supplementary material for: Association of sublingual microcirculation parameters and endothelial glycocalyx dimensions in resuscitated sepsis
Source: Crit Care. 2019 Jul 24;23:260. doi: 10.1186/s13054-019-2542-2 (PMC6657098; doi:10.1186/s13054-019-2542-2)
Supplement: Supplementary file 1 — Table S1. Summary of microcirculation and endothelial glycocalyx parameters [16, 25]. Figure S1. Analysis of the endothelial glycocalyx (eGC) in cell culture via atomic force microscopy (AFM). Surface approach: the cantilever (AFM tip) approaches the sample surface vertically. The reflection of a laser beam from the back of the cantilever is continuously detected by a photodiode. First slope: reaching the surface, the cantilever, serving as a soft spring, is deflected while indenting into the sample. The changing laser beam reflection is plotted as a function of sample position along the z-axis. By including the cantilever’s spring constant and the optical lever sensitivity, a force-versus-indentation curve can be generated to provide information about how much force (in pN) is needed to indent a certain distance (in nm) into the sample. The first slope of the curve reflects the indentation of the eGC. Second slope: in the second slope, more force is needed to indent into the surface, which reflects the cell cortex with the plasma membrane and actin web. Due to the linearity of the first slope, a regression line can be generated manually through the starting points of both slopes using PUNIAS (protein unfolding and nano-indentation analysis software, version 1.0, release 2.1, http://punias.free.fr). Projected to the x-axis, the distance between both starting points represents the thickness of the eGC [38, 39]. Table S2. Baseline characteristics of septic patients stratified for sepsis duration. Figure S2. Endothelial glycocalyx dimensions measured in vivo and in vitro. Scatter plot showing the association between AFM-derived eGC thickness (in vitro) and corresponding PBR values (in vivo) in three apparently healthy individuals (white circles) and in three septic patients (black circles). Each circle represents the mean of three independent experiments (consisting of ≥ 5 indentation curves in each of ≥ 10 cells) for each individual serum. Incubation without human ser [file 13054_2019_2542_MOESM1_ESM.docx]

# **Additional File 1**

**Table S1**: Summary of microcirculation and endothelial glycocalyx parameters [1, 2]

| \| **Variable** \| **Abbreviation** \| **Unit** \| **Definition** \| \| --- \| --- \| --- \| --- \| \| Total vessel density \| TVD \| mm/mm^2^ \| A measure of all vessels over the field of view. Computed as total length of small vessels divided by total area of field of view. \| \| Perfused vessel density \| PVD \| mm/mm^2^ \| A measure of the perfused vessel lineal density. Percentage of perfused vessels × TVD \| \| Proportion of perfused vessels \| PPV \| % \| A measure of the lineal proportion of perfused vessels. Computed as total length of perfused small vessels (semiqualitative velocity score 2 or greater)^1^ divided by total length of small vessels. \| \| Microvascular flow index \| MFI \| Points \| A qualitative assessment of flow in the video. MFI was calculated by scoring all individual vessels on the screen based on their microcirculatory perfusion scoring (0 - absent, 1 - intermittent, 2 - sluggish, 3 - normal) and averaging their scores[3]. \| \| Microvascular flow index (eyeballing) \| MFI (eyeballing) \| Points \| A qualitative assessment of flow over quadrants. Predominant (mode) flow velocity of visualized vessels is determined for each image quadrant. MFI is computed as the average of the predominant flow classification of small vessels over the four image quadrants. \| \| Heterogeneity Index \| HI \| % \| The difference between extreme values of MFI between recordings divided by its mean value \| \| Perfused boundary region \| PBR \| μm \| An inverse parameter of the endothelial glycocalyx dimensions defined as the distance between the RBC column width and the outer edge of the RBC perfused lumen. PBR is calculated automatically by the software (GlycoCheck^TM^). \| |
| --- | --- | --- | --- | --- | --- | --- | --- | --- | --- | --- | --- | --- | --- | --- | --- | --- | --- | --- | --- | --- | --- | --- | --- | --- | --- | --- | --- | --- | --- | --- | --- | --- |

^1^ Semiquantitative velocity scoring: 0 = absent flow; 1 = non-continuous/sluggish flow; 2 = moderate flow; 3 = normal continuous/brisk flow

Abbreviations Table S1:

RBC = Red blood cell

**
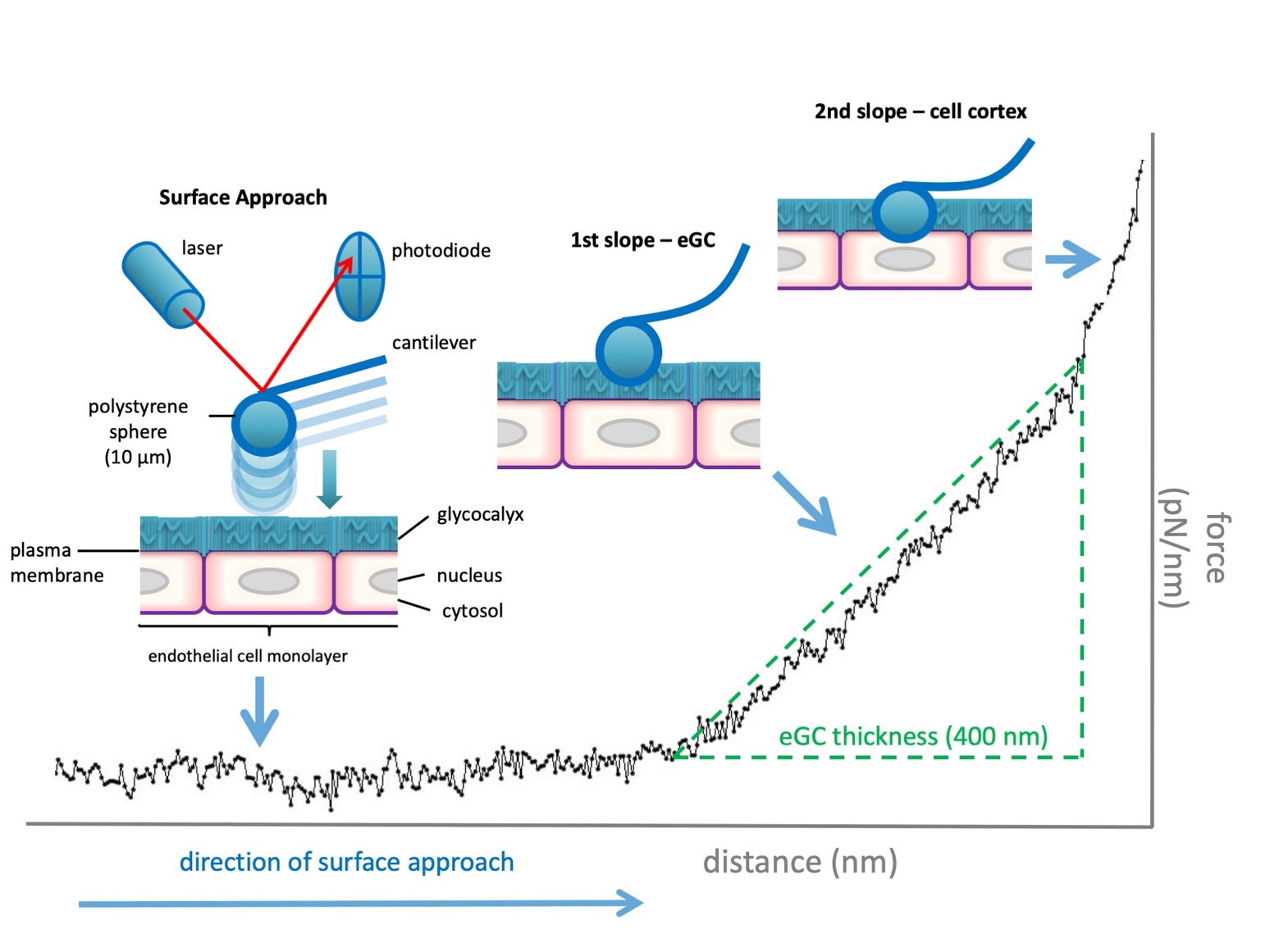
**

**Figure A1: Analysis of the endothelial glycocalyx (eGC) in cell culture via atomic force microscopy (AFM).**

**Surface Approach:** The cantilever (AFM tip) approaches the sample surface vertically. The reflection of a laser beam from the back of the cantilever is continuously detected by a photodiode. **1st slope:** Reaching the surface, the cantilever, serving as a soft spring, is deflected while indenting into the sample. The changing laser beam reflection is plotted as a function of sample position along the z-axis. By including the cantilever’s spring constant and the optical lever sensitivity, a force-versus-indentation curve can be generated to provide information about how much force (in pN) is needed to indent a certain distance (in nm) into the sample. The first slope of the curve reflects the indentation of the eGC. **2nd slope:** In the second slope, more force is needed to indent into the surface, which reflects the cell cortex with plasma membrane and actin web. Due to the linearity of the first slope, a regression line can be generated manually through the starting points of both slopes using PUNIAS (Protein Unfolding and Nano-Indentation Analysis Software, Version 1.0, Release 2.1, http://punias.voila.net/). Projected to the x-axis, the distance between both starting points represents the thickness of the eGC [4, 5].

**Table A2:** Baseline characteristics of septic patients stratified for sepsis duration

| \| **Variable** \| **Septic Patients**  **(Sepsis onset ≤ 24h)** \| **Septic Patients**  **(Sepsis onset >24 h)** \| ***P value*** \| \| --- \| --- \| --- \| --- \| \| Number of participants (n; %) \| 9 \| 21 \| - \| \| Female sex (n; %) \| 3 (33.33) \| 4 (19.05) \| 0.64 \| \| Age (years, median (IQR)) \| 71 (60 – 81) \| 66 (58 – 78) \| 0.53 \| \| BMI (kg/m^2^, median (IQR)) \| 26.8 (21.2 – 31.6) \| 24.9 (21.5 – 27.1) \| 0.60 \| \| SOFA score (median (IQR)) \| 10 (5 – 14) \| 9 (5 – 12) \| 0.82 \| \| Organ replacement therapy (n; %) \| 4 (44.4) \| 14 (66.7) \| 0.42 \| \| Mechanical ventilation (n; %) \| 4 (44.4) \| 13 (61.9) \| 0.44 \| \| Acute dialysis (n; %) \| 0 (0) \| 5 (23.8) \| 0.15 \| \| Vasopressors (n; %) \| 7 (77.8) \| 13 (61.9) \| 0.68 \| \| Norepinephrine (μg/kg/min) \| 0.08 (0.02 – 0.23) \| 0.03 (0 – 0.01) \| 0.18 \| \| Septic shock (n; %)** \| 1 (11.1) \| 2 (9.5) \| 0.99 \| \| Hospital mortality (n; %) \| 2 (22.2) \| 7 (33.3) \| 0.68 \| \| CCI score (median (IQR)) \| 1 (0.5 – 2) \| 2 (0.5 – 3) \| 023 \| \| **Comorbidities** (n; %) \|  \|  \|  \| \| Chronic respiratory disease \| 2 (22.2) \| 7 (33.3) \| 0.99 \| \| Congestive heart failure \| 6 (66.7) \| 10 (47.6) \| 0.44 \| \| Chronic hepatic disease \| 0 (0) \| 3 (30) \| 0.53 \| \| Dialysis-dependent CKD \| 1 (11.1) \| 0 (0) \| 0.30 \| \| Malignancy \| 1 (11.1) \| 4 (19.1) \| 0.99 \| \| Diabetes Mellitus \| 3 (33.3) \| 2 (9.5) \| 0.14 \| \| **Focus of infection** (n; %) \|  \|  \|  \| \| Respiratory tract \| 4 (44.4) \| 13 (61.9) \| - \| \| Prosthesis \| 2 (22.2) \| 2 (9.5) \| - \| \| Gastrointestinal tract \| 1 (11.1) \| 2 (9.5) \| - \| \| Unknown \| 0 (0) \| 2 (9.5) \| - \| \| Skin \| 1 (11.1) \| 1 (4.8) \| - \| \| Urinary tract \| 1 (11.1) \| 0 (0) \| - \| \| Heart \| 0 (0) \| 1 (4.8) \| - \| \| **Endothelial Glycocalyx** (median (IQR)) \|  \|  \|  \| \| PBR 5 – 25 (µm) \| 2.36 (2.27 – 2.44) \| 2.32 (2.19 – 2.49) \| 0.89 \| \| Syndecan-1 (ng/ml) \| 127.1 (90.44 – 347.70) \| 215.3 (135.6 – 366.1) \| 0.26 \| \| **Microcirculation data** (median (IQR)) \|  \|  \|  \| \| TVD (mm/mm^2^) \| 19.92 (19.14 – 20.96) \| 18.70 (16.41 – 20.02) \| 0.07 \| \| PVD (mm/mm^2^) \| 18.35 (17.37 – 20.47) \| 15.66 (12.85 – 18.96) \| 0.03 \| \| PPV (%) \| 95.54 (85.54 – 97.31) \| 91.64 (84.46 – 96.89) \| 0.56 \| \| MFI (points) \| 2.79 (2.61 – 2.91) \| 2.68 (2.44 – 2.92) \| 0.63 \| \| HI (%) \| 0.06 (0.01 – 0.16) \| 0.09 (0.03 – 0.16) \| 0.53 \| \| **Macrocirculation data** (median (IQR)) \|  \|  \|  \| \| MAP (mmHg) \| 70 (65 – 74) \| 77.3 (67.7 – 87) \| 0.11 \| \| Heart Rate (pulse/min) \| 87 (76 – 107) \| 92 (80 – 100) \| 0.63 \| \| Respiratory Rate (breaths/min) \| 18 (16 – 27) \| 21 (17 – 27) \| 0.47 \| \| Temperature (°C) \| 37.7 (36.8 – 38.4) \| 36.7 (36.3 – 37.4) \| 0.07 \| \| **Laboratory data** (median (IQR)) \|  \|  \|  \| \| CRP (mg/dl) \| 22.9 (19.4 – 34.4) \| 23.4 (16.0 – 33.4) \| 0.76 \| \| IL-6 (ng/ml) \| 852 (96 – 4956) \| 347 (96 – 1011) \| 0.14 \| \| PCT (ng/ml) \| 22.10 (1.95 – 233.28) \| 6.34 (1.16 – 48.37) \| 0.48 \| \| pH \| 7.40 (7.36 – 7.42) \| 7.43 (7.36 – 7.49) \| 0.53 \| \| Lactate (mmol/l) \| 2 (1.60 – 2.25) \| 1.60 (0.90 – 1.85) \| 0.09 \| \| Albumin (g/dl) \| 2.6 (1.9 – 3.0) \| 2.4 (2.2 – 2.9) \| 0.82 \| \| Total serum protein (g/dl) \| 5.4 (4.4 – 6.3) \| 5.5 (5.2 – 5.9) \| 0.94 \| |
| --- | --- | --- | --- | --- | --- | --- | --- | --- | --- | --- | --- | --- | --- | --- | --- | --- | --- | --- | --- | --- | --- | --- | --- | --- | --- | --- | --- | --- | --- | --- | --- | --- | --- | --- | --- | --- | --- | --- | --- | --- | --- | --- | --- | --- | --- | --- | --- | --- | --- | --- | --- | --- | --- | --- | --- | --- | --- | --- | --- | --- | --- | --- | --- | --- | --- | --- | --- | --- | --- | --- | --- | --- | --- | --- | --- | --- | --- | --- | --- | --- | --- | --- | --- | --- | --- | --- | --- | --- | --- | --- | --- | --- | --- | --- | --- | --- | --- | --- | --- | --- | --- | --- | --- | --- | --- | --- | --- | --- | --- | --- | --- | --- | --- | --- | --- | --- | --- | --- | --- | --- | --- | --- | --- | --- | --- | --- | --- | --- | --- | --- | --- | --- | --- | --- | --- | --- | --- | --- | --- | --- | --- | --- | --- | --- | --- | --- | --- | --- | --- | --- | --- | --- | --- | --- | --- | --- | --- | --- | --- | --- | --- | --- | --- | --- | --- | --- | --- | --- | --- | --- | --- | --- | --- | --- | --- | --- | --- | --- | --- | --- | --- | --- | --- | --- | --- | --- | --- | --- | --- | --- | --- | --- | --- | --- | --- | --- | --- | --- | --- | --- | --- | --- | --- | --- |

Abbreviations Table A2:

BMI = Body mass index, CCI score = Charlson Comorbidity Index, CKD = Chronic kidney disease, CNS = Central nervous system, CRP = C-reactive protein, HI = Heterogeneity index, IL-6 = Interleukin-6, IQR = interquartile range, MAP = Mean arterial pressure, MFI = Microvascular flow index, PBR = Perfused boundary region, PCT = Procalcitonin, PPV = Proportion of perfused vessels, PVD = Perfused vessel density, RBC = Red blood cell, SOFA score = Sequential Organ Failure Assessment score, TVD = Total vessel density, WBC = White blood cell

**Figure A2: Endothelial glycocalyx dimensions measured *in vivo* and *in vitro*.**

Scatter plot showing the association between AFM-derived eGC thickness (*in vitro*) and corresponding PBR values (*in vivo*) in three apparently healthy individuals (white circles) and in three septic patients (black circles). Each circle represents the mean of three independent experiments (consisting of ≥ 5 indentation curves in each of ≥ 10 cells) for each individual serum. Data are presented as mean ± SEM.

PBR = Perfused boundary region, SEM = Standard error of mean

**Table A3**: Correlations of PBR, PPV, MFI and age, sex, comorbidities in the septic cohort

| \| **Variable** \| **PBR** \| **MFI** \| **PPV** \| \| --- \| --- \| --- \| --- \| \| Age (years) \| -0.79  (0.68) \| -0.14  (0.46) \| 0.19  (0.32) \| \| Sex (female) \| 0.23  (0.91) \| -0.30  (0.11) \| -0.32  (0.08) \| \| CCI (points) \| 0.09  (0.63) \| 0.01  (0.97) \| 0.05  (0.80) \| \| Congestive heart failure \| -0.16  (0.40) \| 0.06  (0.75) \| 0.03  (0.87) \| \| Coronary heart disease \| -0.13  (0.49) \| 0.16  (0.41) \| 0.15  (0.44) \| \| Chronic hepatic disease \| 0.29  (0.12) \| 0.33  (0.08) \| 0.33  (0.07) \| \| Dialysis-dependent CKD \| -0.31  (0.09) \| 0.29  (0.12) \| 0.28  (0.14) \| \| Malignancy \| 0.32  (0.09) \| -0.17  (0.38) \| -0.18  (0.34) \| \| Diabetes Mellitus \| -0.15  (0.45) \| -0.12  (0.51) \| -0.22  (0.25) \| \|  \|  \|  \|  \| |
| --- | --- | --- | --- | --- | --- | --- | --- | --- | --- | --- | --- | --- | --- | --- | --- | --- | --- | --- | --- | --- | --- | --- | --- | --- | --- | --- | --- | --- | --- | --- | --- | --- | --- | --- | --- | --- | --- | --- | --- | --- | --- | --- | --- | --- |

Spearman correlation was used. The p values are indicated in brackets.

Abbreviations Table A2:

CCI = Charlson comorbidity index, CKD = Chronic kidney disease, MFI = Microvascular flow index, PBR = Perfused boundary region 5–25 μm, PPV = proportion of perfused vessels

**Table A4**: Simple and multiple linear regression (PBR as dependent variable)

| Independent Variable | Standardized Coefficient β | *P* value |
| --- | --- | --- |
| *TVD unadjusted* | 0.001 | 0.99 |
| *TVD adjusted for:*  - Age, sex, BMI, CCI, sepsis | 0.13 | 0.58 |
| - SOFA score, septic shock, norepinephrine dose, PCT | 0.22 | 0.28 |
| - Age, sex, BMI, CCI, sepsis, SOFA score, septic shock, norepinephrine dose, PCT | 0.28 | 0.33 |

| Independent Variable | Standardized Coefficient β | *P* value |
| --- | --- | --- |
| *MFI unadjusted* | -0.10 | 0.53 |
| *MFI adjusted for:*  - Age, sex, BMI, CCI, sepsis | 0.12 | 0.63 |
| - SOFA score, septic shock, norepinephrine dose, PCT | 0.34 | 0.31 |
| - Age, sex, BMI, CCI, sepsis, SOFA score, septic shock, norepinephrine dose, PCT | 0.34 | 0.31 |

| Independent Variable | Standardized Coefficient β | *P* value |
| --- | --- | --- |
| *PVD unadjusted* | -0.06 | 0.72 |
| *PVD adjusted for:*  - Age, sex, BMI, CCI, sepsis | 0.09 | 0.71 |
| - SOFA score, septic shock, norepinephrine dose, PCT | 0.28 | 0.22 |
| - Age, sex, BMI, CCI, sepsis, SOFA score, septic shock, norepinephrine dose, PCT | 0.35 | 0.23 |

| Independent Variable | Standardized Coefficient β | *P* value |
| --- | --- | --- |
| *PPV unadjusted* | -0.12 | 0.45 |
| *PPV adjusted for:*  - Age, sex, BMI, CCI, sepsis | 0.09 | 0.71 |
| - SOFA score, septic shock, norepinephrine dose, PCT | 0.28 | 0.22 |
| - Age, sex, BMI, CCI, sepsis, SOFA score, septic shock, norepinephrine dose, PCT | 0.31 | 0.35 |

Dependent Variable: PBR

Abbreviations Table A3:

BMI = Body mass index, CCI = Charlson comorbidity index, MFI = Microvascular flow index, PBR = Perfused boundary region, PCT = Procalcitonin, PPV = Proportion of perfused vessels, PVD = Perfused vessel density, SOFA score = Sequential organ failure assessment score

**Table A5**: Correlations of microcirculatory and eGC parameters in the septic cohort.

|  | **PVD (mm/mm^2^)** | **PPV (%)** | **MFI (pts)** |
| --- | --- | --- | --- |
| **PBR (μm)** | 0.19  (0.33) | 0.19  (0.31) | 0.18  (0.33) |
| **Syndecan-1 (ng/ml)** | -0.04  (0.83) | 0.02  (0.91) | 0.02  (0.90) |

Spearman Correlation was used. The p values are indicated in brackets.

MFI = Microvascular flow index, PBR = Perfused boundary region, PPV = Proportion of perfused vessels, PVD = Perfused vessel density

**Figure A3: Correlations of microcirculation and endothelial glycocalyx parameters.** (A–C): Correlation of PBR with PVD, PPV and MFI. (D–F): Correlation of syndecan-1 with PVD, PPV and MFI.

MFI = Microvascular flow index, PBR = Perfused boundary region, PPV = Proportion of perfused vessels, PVD = Perfused vessel density

**
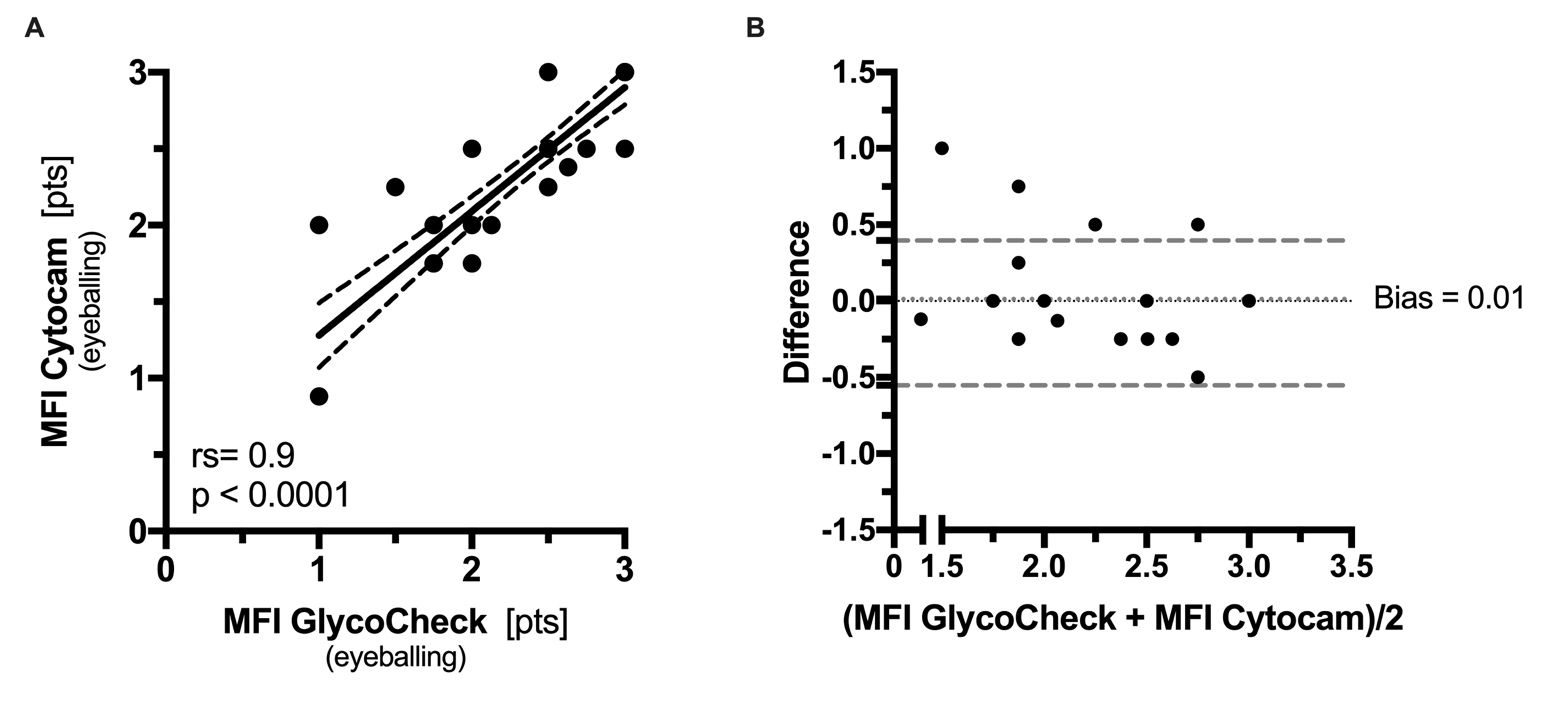
**

**Figure A4: MFI (eyeballing) obtained in real time with the two different systems.** (A): Correlation between the MFI (eyeballing) values obtained at the bedside. (B): Bland–Altman plot showing the limits of agreement (Bias ± 1.96 SD) between paired MFI values for the Cytocam and GlycoCheck system (eyeballing).

One point can represent more than one individual.

MFI = Microvascular flow index, SD = Standard deviation

1. De Backer D, Hollenberg S, Boerma C, Goedhart P, Buchele G, Ospina-Tascon G, Dobbe I, Ince C: **How to evaluate the microcirculation: report of a round table conference**. *Crit Care* 2007, **11**(5):R101.

2. Lee DH, Dane MJ, van den Berg BM, Boels MG, van Teeffelen JW, de Mutsert R, den Heijer M, Rosendaal FR, van der Vlag J, van Zonneveld AJ *et al*: **Deeper penetration of erythrocytes into the endothelial glycocalyx is associated with impaired microvascular perfusion**. *PLoS One* 2014, **9**(5):e96477.

3. Pozo MO, Kanoore Edul VS, Ince C, Dubin A: **Comparison of different methods for the calculation of the microvascular flow index**. *Crit Care Res Pract* 2012, **2012**:102483.

4. Lukasz A, Hillgruber C, Oberleithner H, Kusche-Vihrog K, Pavenstadt H, Rovas A, Hesse B, Goerge T, Kumpers P: **Endothelial glycocalyx breakdown is mediated by angiopoietin-2**. *Cardiovasc Res* 2017, **113**(6):671-680.

5. Oberleithner H, Peters W, Kusche-Vihrog K, Korte S, Schillers H, Kliche K, Oberleithner K: **Salt overload damages the glycocalyx sodium barrier of vascular endothelium**. *Pflugers Arch* 2011, **462**(4):519-528.
